# Assessment of common infections and incident dementia using UK primary and secondary care data: a historical cohort study

**DOI:** 10.1016/S2666-7568(21)00118-5

**Published:** 2021-07

**Authors:** Rutendo Muzambi, Krishnan Bhaskaran, Liam Smeeth, Carol Brayne, Nish Chaturvedi, Charlotte Warren-Gash

**Affiliations:** a Faculty of Epidemiology and Population Health, London School of Hygiene & Tropical Medicine, London WC1E 7HT, UK; b Cambridge Institute of Public Health, University of Cambridge, Cambridge, UK; c Medical Research Council Unit for Lifelong Health and Ageing at University College London, Institute of Cardiovascular Science, University College London, London, UK

## Abstract

**Background:**

Common infections have been associated with dementia risk; however, evidence is scarce. We aimed to investigate the association between common infections and dementia in adults (≥65 years) in a UK population-based cohort study.

**Methods:**

We did a historical cohort study of individuals who were 65 years and older with no history of dementia or cognitive impairment using the Clinical Practice Research Datalink linked to Hospital Episode Statistics between Jan 1, 2004, and Dec 31, 2018. Multivariable Cox proportional hazard regression models were used to estimate the association between time-updated previous common infections (sepsis, pneumonia, other lower respiratory tract infections, urinary tract infections, and skin and soft tissue infections) and incident dementia diagnosis. We also tested for effect modification by diabetes since it is an independent risk factor for dementia and co-occurs with infection.

**Findings:**

Between Jan 1, 2004, and Dec 31, 2018, our study included 989 800 individuals (median age 68·6 years [IQR 65·0–77·0]; 537 602 [54·3%] women) of whom 402 204 (40·6%) were diagnosed with at least one infection and 56 802 (5·7%) had incident dementia during a median follow-up of 5·2 years (IQR 2·3–9·0). Dementia risk increased in those with any infection (adjusted hazard ratio [HR] 1·53 [95% CI 1·50–1·55]) compared with those without infection. HRs were highest for sepsis (HR 2·08 [1·89–2·29]) and pneumonia (HR 1·88 [1·77–1·99]) and for infections leading to hospital admission (1·99 [1·94–2·04]). HRs were also higher in individuals with diabetes compared with those without diabetes.

**Interpretation:**

Common infections, particularly those resulting in hospitalisation, were associated with an increased risk of dementia persisting over the long term. Whether reducing infections lowers the risk of subsequent dementia warrants evaluation.

**Funding:**

Alzheimer's Society, Wellcome Trust, and the Royal Society.

## Introduction

Dementia (also known as major neurocognitive disorder) is a leading contributor to disability and dependence worldwide. In the UK, the number of people living with dementia is projected to rise from 850 000 in 2015 to more than 2 million by 2051, owing to population growth and ageing.^1^ Because the established modifiable risk factors of dementia account for only 40% of dementia cases, identification of other preventable risk factors has become an urgent public health priority.^2^

Common infections are an established risk factor for acute cognitive impairment in older adults (≥65 years) and increasing evidence from longitudinal studies suggests that common infections are associated with an increased risk of dementia.^3^ However, previous studies have mainly focused on sepsis or pneumonia hospitalisations rather than on a wide range of infections in different clinical settings,^4^, ^5^, ^6^, ^7^, ^8^ and have had short follow-up periods (3–5 years),^4^, ^7^ relatively small sample sizes (between 3069 and 5955 participants or cases),^5^, ^6^, ^7^ or inadequate adjustment for confounding.^6^, ^7^, ^8^ Large-scale longitudinal studies published since 2016 have overcome some of these limitations; however, these studies have only been done in veterans,^9^ stroke survivors,^10^ or patients in intensive care unit,^11^ restricting the generalisability of their findings. Furthermore, evidence of an association between infections and dementia risk is conflicting.^11^ Moreover, few studies have assessed the effect of type, clinical setting, and frequency of infections on dementia risk, restricting the potential for public health intervention.

Serious infections, including sepsis, infections requiring hospitalisation or infections resulting in mortality, frequently occur in older adults (≥70 years) with diabetes.^12^ Diabetes is an independent risk factor for dementia,^13^ and, given its co-occurrence with infections, diabetes could potentially modify the association between common infections and dementia; however, to our knowledge, no studies have explored this link.

**Research in context**

**Evidence before this study**

We updated our previously published systematic review of longitudinal studies investigating the association between common bacterial infections and dementia or cognitive decline by searching Embase and MEDLINE for articles published between March 1, 2019, and Dec 22, 2020, without language or geographical location restrictions. We used medical subject heading terms and key words including “dementia”, “cognitive decline”, “sepsis”, “lower respiratory tract infections”, “urinary tract infections”, and “skin and soft tissue infections.” A full list of search terms is reported in the appendix (pp 1–4). Our updated search yielded two new studies. Overall, most of the identified studies, which were mainly done in the USA or Taiwan, suggested an association between common infections and dementia. One study did not find an association; however, this study was done only in patients in an intensive care unit setting, restricting the generalisability of these findings. Other studies also had limitations, including short follow-up periods, relatively small sample sizes, or inadequate adjustment for confounders. Additionally, previous studies mainly focused on sepsis or pneumonia hospitalisations. Two studies also investigated the effect of frequency of infections on dementia, and only one investigated the effect of clinical setting of infections on dementia.

**Added value of this study**

To our knowledge, our longitudinal study of almost 1 million individuals aged 65 years and older, with up to 14 years of follow-up, is the largest to date reporting on the association between common infections and incident dementia. Our study finds novel evidence of effect modification by diabetes on the association between infections and dementia. Our finding of a higher dementia risk for infections likely to be severe, such as hospitalised infections, sepsis, and pneumonia, also extends the existing literature as does our finding that the association between infections and dementia persists continues long term. We also confirm findings from previous longitudinal population-based studies in the USA and Taiwan that sepsis and pneumonia are associated with incident dementia, and findings from a US study of predominantly male veterans and a UK study of stroke survivors, which also showed associations between urinary tract infections and skin and soft tissue infection and incident dementia.

**Implications of all the available evidence**

Our findings highlight the need for future studies investigating whether reducing infections lowers the risk of subsequent dementia. More work is needed to understand the underlying mechanisms in the association between infections and dementia and to investigate the association between common infections and cognitive decline using validated repeated measures of cognition.

We aimed to investigate the association between common infections and dementia or cognitive impairment in a large population-based cohort study of adults aged 65 years and older using UK electronic health records data. We hypothesised that all common infections, regardless of type of infection (sepsis, pneumonia, other lower respiratory tract infections, urinary tract infections, and skin and soft tissue infections), clinical setting, number of infections, and time since infection, increased the risk of dementia and that diabetes modified the association between infections and dementia.

## Methods

### Study design and participants

In this historical cohort study, we used data from the Clinical Practice Research Datalink (CPRD) GOLD linked to secondary care data from the Hospital Episode Statistics (HES). CPRD is a large primary care database of anonymous electronic health records representative of the UK population in terms of age, sex, and ethnicity, and it includes information on demographics, diagnoses, therapies, and referrals.^14^ The HES database includes data on admissions to English National Health Service hospitals and independent health-care providers.^15^ Clinical diagnoses in CPRD were coded using Read, and diagnoses with HES were coded with International Classification of Diseases (ICD)-10.

We included adults aged 65 years and older present in CPRD GOLD with linked HES data who were registered in CPRD between Jan 1, 2004, and Dec, 31, 2018. Only individuals who were registered in CPRD at least 12 months before the start of follow-up and who had no history of dementia or cognitive impairment were included. Follow-up began on Jan 1, 2004, participants' 65th birthday, or 12 months after registration in CPRD, whichever occurred first, and participants were followed up to the earliest incident dementia diagnosis, date of death, deregistration from CPRD, the last data collection date by the general practitioner, or end of the study period, whichever occurred first. To avoid misclassifying delirium, which often results from infection and is characterised by acute cognitive dysfunction and inattention, as dementia, we excluded the first 3 months of follow-up after infection.

### Procedures

Our primary exposure was a time-updated variable capturing whether an individual had ever had a recorded common infection during follow-up. Exposure status changed after diagnosis of infection. Therefore, individuals initially contributed person-time to the unexposed category (no infection) but once diagnosed with an infection they contributed person-time to the exposed category (infection). These infections included sepsis, pneumonia, other lower respiratory tract infections, urinary tract infections, and skin and soft tissue infections. In HES, ICD-10 codes were used to define all infections. In CPRD, sepsis, pneumonia, and other lower respiratory tract infections were defined using read codes; individuals were defined as having urinary tract infections or skin and soft tissue infections if they had a Read code and a prescription for antibiotics on the same date. In our secondary analyses, we explored the effect of type, clinical setting (hospital-recorded or general practitioner-recorded), frequency, and timing of infections on dementia incidence. Time since infection was split into overlapping periods (3 months to <1 year, 3 months to <2 years, and 3 months to <3 years, 3 months to <4 years, 3 months to <5 years, 3 months to <6 years, 3 months to <7 years, 3 months to <8 years, 3 months to <9 years, and 3 months to ≥9 years). The association between infections and dementia was assessed in these time periods. We considered infections that occurred within 28 days of each other as a single episode of infection.

We used a directed acyclic graph to identify potential confounders and mediators of the association between infections and dementia (appendix p 5). Potential confounders included age (years), sex (male and female), ethnicity (White, south Asian, Black, and mixed ethnicity or other), time updated calendar period (split into 2004–08, 2009–13, or 2014–18) to account for clinical and administrative changes over the study period that might influence the recording of infections and dementia diagnoses in our study, patient-level quintiles of the Index of Multiple Deprivation (IMD) as a proxy for socioeconomic status, heavy alcohol consumption, smoking status (current smoker, former smoker, or never-smoked) and body-mass index (BMI; according to WHO categories). We identified the following comorbidities as potential confounders at any time before the start of follow-up: severe mental illness (schizophrenia and bipolar disorder), depression and anxiety, inflammatory bowel disease, multiple sclerosis, rheumatoid arthritis, psoriasis, hypertension, heart failure, type 1 and type 2 diabetes mellitus, chronic kidney disease, chronic liver disease, chronic obstructive pulmonary disease, obstructive sleep apnoea, stroke, and traumatic brain injury. The following covariates relating to medication use were identified in the 2 years before the start of follow-up: benzodiazepines, proton pump inhibitors, corticosteroids, and polypharmacy (concurrent use of five or more medications). Diabetes was also identified as an effect modifier. Atrial fibrillation, stroke, and myocardial infarction were identified as potential mediators and were measured after infection diagnosis. The ascertainment of variables is reported in the appendix (pp 6–9).

### Outcomes

Our primary outcome was an incident dementia diagnosis. When individuals were diagnosed with dementia in both CPRD and HES, we used the earliest dementia diagnosis. We used a broad definition of dementia that included Alzheimer's disease, vascular dementia, unspecified dementia, and secondary dementia related to other diseases. Our secondary outcome was cognitive impairment, which was defined using Read and ICD-10 codes relating to symptoms and diagnoses of cognitive impairment.

### Statistical analysis

Our statistical analysis plan was specified in the study protocol before doing our analyses. We described the study characteristics of individuals with and without common infections during follow-up and examined age-specific incidence rates of dementia in person-time with and without common infections.

We used Cox proportional hazards regression, with age as the underlying timescale, to calculate hazard ratios (HRs) and 95% CI to investigate the association between common infections and risk of dementia. In our minimally adjusted model, we adjusted for age, sex, patient-level IMD, and calendar period. In our fully adjusted model, we adjusted for age, sex, patient-level IMD, calendar period, ethnicity, smoking status, heavy alcohol consumption, anxiety and depression, severe mental illness, inflammatory bowel disease, multiple sclerosis, rheumatoid arthritis, psoriasis, asthma, chronic kidney disease, chronic liver disease, chronic obstructive pulmonary disease, diabetes mellitus, heart failure, hypertension, obstructive sleep apnoea, stroke, traumatic brain injury, benzodiazepines, proton pump inhibitors, systemic corticosteroids, and polypharmacy. BMI was not adjusted for in our main analysis because a low BMI might be a consequence of dementia. However, we explored the possible confounding effect of BMI in an additional analysis. We adjusted for potential mediators (atrial fibrillation, stroke, and myocardial infarction) in separate analyses. A complete case analysis approach was used when HRs were adjusted for BMI, ethnicity, and smoking status. We did not do multiple imputation because these data were unlikely to be missing at random.^16^

To test the robustness of our findings, we did a range of sensitivity analyses (appendix p 10). First, we repeated our primary analyses excluding individuals with secondary dementia causally related to other conditions because infections are unlikely to be causally associated with this type of dementia. Second, to improve the accuracy of our definition of cognitive impairment, we excluded codes relating to symptoms of cognitive impairment. Third, we repeated our primary analyses defining all infections in CPRD with a diagnostic code and prescription for antibiotics to improve the accuracy of our infection definition. Finally, we repeated our primary analysis excluding individuals diagnosed with infections from two different sites (eg, skin and soft tissue infections and pneumonia) on the same date to avoid the potential biases introduced from including these infections.

A p value of less than 0·05 was considered to be statistically significant.

Using Cox regression models, we assessed the effect of type, clinical setting, frequency, and timing of infections on the risk of dementia. For our analyses on timing of infections, follow-up was split into overlapping periods to account for depletion of susceptible bias.^17^ We tested for the presence of effect modification by diabetes using the likelihood ratio test. For our secondary outcome, we explored the effect of common infections on cognitive impairment.

We did three additional secondary analyses: (1) we investigated whether the association between infections and dementia differed across subtypes of dementia (Alzheimer's disease, vascular dementia, and unspecified dementia); (2) we assessed whether the association between infections and dementia varied by sex; and (3) we used Kaplan-Meir survival plots and the log-rank test to explore proximity to death after dementia diagnosis in people with and without infections, given that serious cognitive impairment can occur in the phase before death.

We tested the Cox proportional hazards assumption using log-log plots and the Schoenfeld residuals test. We found evidence of non-proportionality for our infection variable, suggesting that the association between infections and dementia differed with age. To deal with this, we did additional analyses in which we stratified our primary analyses by age. Other variables showed evidence of non-proportionality (appendix p 22), so we did additional analyses with interaction terms between age and these variables to check whether the observed association between infections and dementia differed from the association in our primary analyses.

Our study was approved by the Independent Scientific Advisory Committee (approval 19_129R) and the London School of Hygiene & Tropical Medicine (reference 17752). Our study was reported in accordance with the Strengthening the Reporting of Observational Studies in Epidemiology guideline. Analyses were done with STATA MP (version 16) and the analysis codes are available online.

### Role of the funding source

The funders had no role in the study design, collection, analysis and interpretation of the data, or writing of the report. The corresponding author (RM) had full access to all the data in the study and accepts responsibility for the decision to submit for publication.

## Results

Between Jan 1, 2004, and Dec, 31, 2018, we included a total of 989 800 individuals in our final study population (appendix p 11), with a median follow-up of 5·2 years (IQR 2·3–9·0). Median age was 68·6 years (65·0–77·0), and 537 602 (54·3%) participants were women (table 1).

**Table 1:** Baseline characteristics

		**Patients (n=989 800)**
Length of CPRD follow-up in years		5·2 (2·3–9·0)
Mean age, years		71·7 (7·9)
Median age, years		68·6 (65·0–77·0)
Age groups, years		
	65–69	528 580 (53·4%)
	70–74	153 969 (15·6%)
	75–79	124 514 (12·6%)
	80–84	97 505 (9·9%)
	85–89	50 785 (5·1%)
	≥90	34 447 (3·5%)
Sex		
	Male	452 198 (45·7%)
	Female	537 602 (54·3%)
Ethnicity		
	White	865 338 (87·4%)
	South Asian	16 901 (1·7%)
	Black	8749 (0·9%)
	Mixed/Other	9938 (1·0%)
	Missing	88 874 (9·0%)
Patient-level Index of Multiple Deprivation		
	1 (least deprived)	238 449 (24·1%)
	2	233 517 (23·6%)
	3	213 430 (21·6%)
	4	172 086 (17·4%)
	5 (most deprived)	132 318 (13·4%)
Body-mass index		
	Underweight (<18·5)	17 480 (1·8%)
	Normal weight (18·5–24·9)	313 044 (31·6%)
	Overweight (25·0–29·9)	356 014 (36·0%)
	Obese or morbidly obese (≥30·0)	210 051 (21·2%)
	Missing	93 211 (9·4%)
Smoking status		
	Never-smoked	441 851 (44·6%)
	Current smoker	149 104 (15·1%)
	Former smoker	374 654 (37·9%)
	Missing	24 191 (2·4%)
Heavy alcohol consumption		54 663 (5·5%)
Comorbidities		
	Depression or anxiety	109 672 (11·1%)
	Severe mental illness	7659 (0·8%)
	Inflammatory bowel disease	31 971 (3·2%)
	Multiple sclerosis	3168 (0·3%)
	Rheumatoid arthritis	20 486 (2·1%)
	Psoriasis	37 872 (3·8%)
	Asthma	120 429 (12·2%)
	Chronic kidney disease	40 220 (4·1%)
	Chronic liver disease	18 609 (1·9%)
	Chronic obstructive pulmonary disease	109 199 (11·0%)
	Diabetes	118 148 (11·9%)
	Heart failure	50 976 (5·2%)
	Hypertension	424 439 (42·9%)
	Myocardial infarction	55 590 (5·6%)
	Obstructive sleep apnoea	7278 (0·7%)
	Stroke	44 430 (4·5%)
	Traumatic brain injury	10 692 (1·1%)
Medication use		
	Benzodiazepines	42 066 (4·2%)
	Proton pump inhibitors	191 895 (19·4%)
	Systemic corticosteroids	85 594 (8·6%)
	Polypharmacy	296 201 (29·9%)

Data are n (%), mean (SD), or median (IQR). Comorbidities were assessed at any time before the start of follow-up. Medication use was captured in the 12 months prior to baseline. Polypharmacy as the concurrent use of five or more medications using British National Formulary chapters. CPRD=clinical practice research datalink.

402 204 (40·6%) individuals were diagnosed with a first-ever infection during the study. Of these individuals, 6046 (1·5%) had sepsis, 16 391 (4·1%) had pneumonia, 197 823 (49·2%) had other lower respiratory tract infections, 110 759 (27·5%) had urinary tract infections, and 68 222 (17·0%) had skin and soft tissue infections. 2488 (0·6%) individuals were diagnosed with multiple infections, other than sepsis, at different sites on the same date. 56 802 (5·7%) of 989 800 individuals had a first-ever diagnosis of dementia during follow-up. The age-specific incidence rate of dementia was higher per person-time with previous infections compared with person-time without previous infections (appendix p 12). Of the individuals diagnosed with dementia, the mean time from an infection diagnosis to a first-ever dementia diagnosis was 4·3 years (SD 3·5) and the median time was 3·7 years (IQR 1·7–6·4). For individuals without an infection diagnosis, the mean time from the start of follow-up to dementia diagnosis was 4·8 years (SD 3·5) with a median time of 4·1 years (IQR 1·8–7·2).

A history of any infection was associated with an increased risk of subsequent dementia compared with no history of infection in our age-adjusted models (HR 1·78 [95% CI 1·75–1·81]; table 2). After fully adjusting for potential confounders, the association between any infection and dementia risk attenuated (HR 1·53 [1·50–1·55]). In the fully adjusted analysis, when we stratified our primary analysis by type of infection, dementia risk was highest for individuals previously infected with sepsis (HR 2·08 [1·89–2·29]) and for individuals with previous pneumonia (HR 1·88 [1·77–1·99]; table 2). Multiple infections diagnosed from different sites on the same date were strongly associated with dementia risk (HR 2·68 [2·40–2·99]). After adjustment for potential mediators, the risk of dementia following infections increased; adjustment for BMI did not change our effect estimates (appendix p 13).

**Table 2:** Association between common infections and dementia overall and stratified by type of infection

	**Total number of incident dementia diagnoses**	**Total person-years at risk**	**Crude incidence rate (95% CI)**	Age-adjusted HR (95% CI)*	**Age, sex, IMD, and calendar period adjusted HR (95% CI)**†	**Fully adjusted HR (95% CI)**‡
No infection	25 314	3 895 032	6·50 (6·42–6·58)	1 (ref)	1 (ref)	1 (ref)
Any infection	31 488	1 754 956	17·94 (17·75–18·14)	1·78 (1·75–1·81)	1·64 (1·61–1·66)	1·53 (1·50–1·55)
Sepsis	427	16 814	25·40 (23·10–27·92)	2·48 (2·26–2·73)	2·18 (1·98–2·40)	2·08 (1·89–2·29)
Pneumonia	1247	47 836	26·07 (24·66–27·56)	2·27 (2·15–2·41)	1·98 (1·87–2·10)	1·88 (1·77–1·99)
Other LRTI	13 429	910 432	14·75 (14·50–15·00)	1·57 (1·54–1·60)	1·39 (1·36–1·42)	1·34 (1·31–1·37)
UTI	10 513	481 341	21·84 (21·43–22·26)	2·04 (1·99–2·08)	1·80 (1·75–1·84)	1·73 (1·69–1·78)
SSTI	5535	291 603	18·98 (18·49–19·49)	1·78 (1·73–1·83)	1·58 (1·53–1·62)	1·54 (1·49–1·58)

HR=hazard ratio. IMD=Index of multiple deprivation. LRTIs=lower respiratory tract infections (excluding pneumonia). UTIs=urinary tract infection. SSTI=skin and soft tissue infection.

* Age as underlying timescale.

† Adjusted for age, sex, patient level IMD, and calendar time period over follow-up (2004–08, 2009–13, and 2014–18).

‡ Adjusted for age, sex, patient level IMD, and calendar time period over follow-up (2004–08, 2009–13, and 2014–18), ethnicity, smoking status, heavy alcohol consumption, anxiety and depression, severe mental illness, inflammatory bowel disease, multiple sclerosis, rheumatoid arthritis, psoriasis, asthma, chronic kidney disease, chronic liver disease, chronic obstructive pulmonary disease, diabetes mellitus, heart failure, hypertension, obstructive sleep apnoea, stroke, traumatic brain injury, benzodiazepines, proton pump inhibitors, systemic corticosteroids, and polypharmacy.

Hospital-recorded infections were associated with an increased risk of dementia (HR 1·99 [95% CI 1·94–2·04; table 3). This association attenuated, but remained strong, when adjusted to only consider hospital-recorded primary diagnosis of infection (HR 1·84 [1·78–1·91]). Evidence of an association for infections recorded in general practice was weak (HR 1·02 [1·00–1·04]).

**Table 3:** Association between common infections and dementia stratified by clinical setting

	**Total number of incident dementia diagnoses**	**Total person-years at risk**	**Crude incidence rate (95% CI)**	Age-adjusted HR (95% CI)*	**Age, sex, IMD, and calendar period adjusted HR (95% CI)**†	**Fully adjusted HR (95% CI)**‡
**General practitioner recorded infections**						
No infection	37 298	4 115 228	9·06 (8·97–9·16)	1 (ref)	1 (ref)	1 (ref)
Any infection	24 314	1 554 615	15·64 (15·44–15·84)	1·20 (1·18–1·22)	1·09 (1·07–1·11)	1·02 (1·00–1·04)
**Hospital recorded infections**						
No infection	51 127	5 534 732	9·24 (9·16–9·32)	1 (ref)	1 (ref)	1 (ref)
Any infection	7166	200 320	35·77 (34·95–36·61)	2·28 (2·22–2·34)	2·15 (2·10–2·20)	1·99 (1·94–2·04)
**Hospital recorded infections (primary diagnosis)**						
No infection	57 142	5 641 094	10·13 (10·05–10·21)	1 (ref)	1 (ref)	1 (ref)
Any infection	3630	10 4147	34·85 (33·74–36·01)	2·10 (2·03–2·17)	1·99 (1·92–2·06)	1·84 (1·78–1·91)

HR=hazard ratio. IMD=Index of multiple deprivation.

* Age as underlying timescale.

† Adjusted for age, sex, patient level IMD, and calendar time period over follow-up (2004–08, 2009–13, and 2014–18).

‡ Adjusted for age, sex, patient level IMD, and calendar time period over follow-up (2004–08, 2009–13, and 2014–18), ethnicity, smoking status, heavy alcohol consumption, anxiety and depression, severe mental illness, inflammatory bowel disease, multiple sclerosis, rheumatoid arthritis, psoriasis, asthma, chronic kidney disease, chronic liver disease, chronic obstructive pulmonary disease, diabetes mellitus, heart failure, hypertension, obstructive sleep apnoea, stroke, traumatic brain injury, benzodiazepines, proton pump inhibitors, systemic corticosteroids, and polypharmacy.

There was a small association between an increasing number of infections and an increasing risk of dementia (HR 1·02 [95% CI 1·01–1·02]; likelihood ratio test for trend p<0·0001; table 4). The risk of dementia was highest between 3 months and 1 year after an infection (HR 1·86 [1·80–1·92]; figure). Dementia risk attenuated over longer periods of follow-up, but risk remained high up to 9 years or more after infection (HR 1·53 [1·50–1·55]). This is also depicted in non-overlapping periods (appendix p 14).

**Table 4:** Association between the number of common infections and dementia

	**Total number of incident dementia diagnoses**	**Total person-years at risk**	Age-adjusted HR (95% CI)*	**Age, sex, IMD, and calendar period adjusted HR (95% CI)**†	**Fully adjusted HR (95% CI)**‡
No infection	25 314	3 895 425	1 (ref)	1 (ref)	1 (ref)
First infection	11 209	859 035	1·52 (1·49–1·55)	1·42 (1·39–1·45)	1·34 (1·32–1·37)
Second and additional infections§	14 112	731 026	1·04 (1·03–1·04)¶	1·02 (1·02–1·03)¶	1·02 (1·01–1·02)¶

HR=hazard ratio. IMD=Index of multiple deprivation.

* Age as the underlying timescale.

† Adjusted for age, sex, patient level IMD, and calendar time period over follow-up (2004–08, 2009–13, and 2014–18).

‡ Adjusted for age, sex, patient level IMD, and calendar time period over follow-up (2004–08, 2009–13, and 2014–18), ethnicity, smoking status, heavy alcohol consumption, anxiety and depression, severe mental illness, inflammatory bowel disease, multiple sclerosis, rheumatoid arthritis, psoriasis, asthma, chronic kidney disease, chronic liver disease, chronic obstructive pulmonary disease, diabetes mellitus, heart failure, hypertension, obstructive sleep apnoea, stroke, traumatic brain injury, benzodiazepines, proton pump inhibitors, systemic corticosteroids, and polypharmacy.

§ Quantitative variable of number of infections from a count of two or more infections alongside a binary variable for yes or no infections in all models. Likelihood ratio test for trend, p<0·0001 in fully adjusted model.

¶ Per additional infection.

**Figure:**
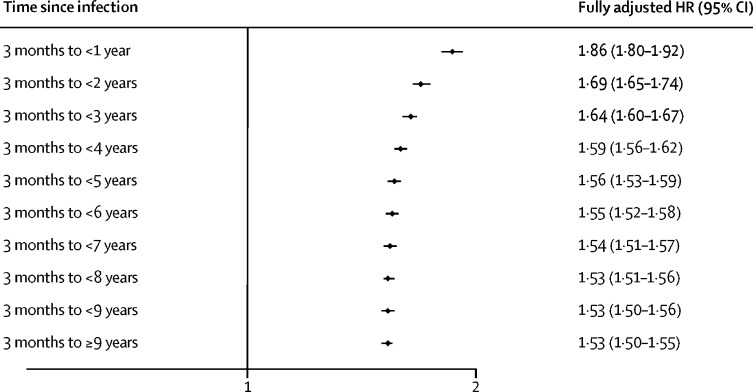
The association between common infections and dementia, stratified according to time since infection Time periods are overlapping. Adjusted for age, sex, patient level index of multiple deprivation, calendar period, ethnicity, smoking status, heavy alcohol consumption, anxiety and depression, severe mental illness, inflammatory bowel disease, multiple sclerosis, rheumatoid arthritis, psoriasis, asthma, chronic kidney disease, chronic liver disease, chronic obstructive pulmonary disease, diabetes mellitus, heart failure, hypertension, obstructive sleep apnoea, stroke, traumatic brain injury, benzodiazepines, proton pump inhibitors, systemic corticosteroids, and polypharmacy. HR=hazard ratio.

There was evidence of effect modification by diabetes on the association between common infections and dementia (p=0·00014; appendix p 15), with a higher risk of dementia in individuals with diabetes (HR 1·70 [95% CI 1·61–1·79]) compared with those without diabetes (HR 1·50 [1·47–1·53]; appendix p 15). We found an association between common infections and an increased risk of cognitive impairment (HR 1·29 [1·27–1·32]; appendix p 16), but this association was weaker than that between infections and dementia (appendix p 16).

Stratifying our main analyses by dementia subtype showed that the association between infections and dementia was highest for unspecified dementia (HR 1·72 [95% CI 1·67–1·76]) and vascular dementia (HR 1·69 [1·62–1·76]), but was markedly weaker for Alzheimer's disease (HR 1·09 [1·05–1·13]; appendix p 17). Stratifying our primary analyses by sex showed that the association between infections and risk of dementia was stronger in men than in women; however, CIs overlapped (appendix p 18). Finally, analyses exploring the proximity to death following a dementia diagnosis show that Kaplan-Meier survival curves were steepest shortly after a dementia diagnosis, indicating that a larger proportion of people died in this period (appendix p 19). The curves also show that survival was worse in those with a history of infections compared with those without a history of infections (p<0·0001).

Due to evidence of non-proportionality for our main infection variable and residual non-proportionality (Schoenfeld residuals test, p = 0·02 and p<0·0001), we stratified our primary analyses by age (appendix pp 20–21). The risk of dementia following infections increased with age, with the risk highest in those aged 90 years and older. When we included interaction terms between age and other variables that showed evidence of non-proportionality, our results were consistent with our primary analysis (appendix p 22).

In our sensitivity analyses, we observed minimal change in our effect estimates for our analyses on infections and dementia (appendix pp 23–24). When we excluded symptoms of cognitive impairment, the magnitude of the association between infections and cognitive impairment diagnoses increased (HR 1·62 [95% CI 1·56–1·68]; appendix p 25).

## Discussion

In this population-based study of almost 1 million adults aged 65 years and older, we found that common infections were associated with an increased risk of dementia, with the risk strongest risk following sepsis and pneumonia. Of note, infections resulting in hospital admission—therefore, probably being more severe—were associated with approximately double the risk of dementia compared with no infections, whereas a weak association was found for infections treated in community general practice. The risk of dementia was strongest between 3 months and 1 year following infection, which might be due to reverse causality (undiagnosed dementia increasing risk of infection); however, the association persisted for more than 9 years. Dementia risk increased with an increasing number of infections, although the magnitude of this trend was small. Furthermore, we found evidence that the association between infection and subsequent dementia was stronger in those with diabetes.

To our knowledge, with almost 1 million individuals followed up for up to 14 years, our study is the largest to investigate the association between common infections and incident dementia. Other strengths of our study include the exclusion of the first 3 months of follow-up after infection to prevent misclassification of delirium as dementia and the use of linked primary and secondary care electronic health records, representative of the English population and thus probably generalisable to the English population aged 65 years and older. Our findings were also robust across a range of sensitivity analyses.

Consistent with our findings, previous longitudinal studies have found an association between sepsis,^4^, ^5^, ^7^, ^8^, ^9^ pneumonia,^5^, ^6^, ^9^ urinary tract infections,^9^, ^10^ skin and soft tissue infections,^9^, ^10^ with dementia. Only two studies investigated the effect of a range of infections on dementia: a US cohort study of 417 172 veterans and a UK cohort study using primary and secondary care data of 60 392 stroke survivors. These studies found that risk of dementia increased with increasing number of infections,^9^, ^10^ and was highest in individuals with sepsis^9^ and for infections requiring hospitalisation.^10^ To our knowledge, only one study did not show an association between infection and dementia: a retrospective Swedish study of 210 334 patients (≥18 years) who had been admitted to an intensive care unit, which found no association between sepsis and dementia.^11^ Discrepancies between the findings of the Swedish study and the rest of the published literature might be due to differences in clinical setting and study population. Similarly, our findings were in contrast with a longitudinal study from the USA that found no risk of cognitive impairment in individuals hospitalised due to sepsis compared with individuals without infection hospitalisation. However, another USA longitudinal study found an association between pneumonia hospitalisation and moderate-to-severe cognitive impairment. In this study, no association was found when individuals hospitalised with pneumonia were compared with individuals who were hospitalised with myocardial infarction or stroke. However, these studies were limited by small sample sizes (both studies included <2402 patients) or unsuitable comparator groups.^18^, ^19^ Our finding of a higher dementia risk in patients aged 80 years and older is in agreement with a case-control study using UK primary care data that reported a stronger association of infections and dementia in adults aged 84 years and older compared with younger age groups.^20^ However, in this study infections were captured only 4 years before a dementia diagnosis. By contrast, a retrospective Taiwanese cohort study reported a lower risk of dementia following sepsis in individuals aged 65 years and older compared with those younger than 65 years. However, confidence intervals in the younger age groups were wide and the number of individuals with dementia was small.

Our study expands on existing literature by examining the association between infections and dementia using a wide range of secondary analyses, including exploring effect modification by diabetes and investigating the association between common infections and cognitive impairment, after resolution of delirium preventing misclassifying delirium as dementia.

The underlying mechanisms driving the association between infections and dementia are unclear, but they might be partly explained by systemic inflammation. Infections trigger the release of proinflammatory cytokines resulting in systemic inflammation, with such inflammation associated with cognitive decline and dementia.^21^, ^22^ Our finding of a larger effect on dementia risk of infections resulting in hospitalisation, such as pneumonia and sepsis, supports the role for notion of more severe infections having a stronger association with dementia risk. Severe infections are more likely to lead to systemic inflammation and are more prevalent in patients with diabetes due to immune dysfunction, probably caused by hyperglycaemia. Systemic inflammation has also been proposed as one of the potential pathways linking diabetes and dementia.^23^ Common infections, particularly respiratory tract infections, have been shown to trigger acute cardiovascular events, including myocardial infarction and stroke, and cause subclinical vascular damage in association with increased inflammation.^24^ This increased inflammation in turn has been associated with vascular dementia.

We found marked differences between the association of common infections and dementia by dementia subtype, with the risk highest for vascular dementia and weakest for Alzheimer's disease. However, caution must be applied when interpreting the weaker associations found for Alzheimer's disease because misclassification of dementia subtype is probable because of the clinical challenges in classifying dementia. Additionally, in older adults, dementia is predominantly associated with mixed pathologies consistent with both vascular dementia and Alzheimer's disease.^25^ Nonetheless, the substantial differences in the strengths of associations between common infections, vascular dementia, and Alzheimer's disease suggest that there might be underlying distinctions between dementia subtypes. Furthermore, mechanisms potentially linking infections with vascular dementia, such as potentiating vascular damage and inflammation, could explain the higher risk observed for vascular dementia.

We found that the risk of dementia following infection was higher in older age groups (≥90 years). This finding might be explained by immune dysfunction, which increases with age. Older adults (≥65 years) are more susceptible to infections and might experience recurrent and more severe infections. The effects of infections on systemic inflammation or vascular damage might accumulate over time, resulting in a higher risk of dementia in older adults. An alternative explanation could be that older adults often have multiple comorbidities and might, therefore, encounter health-care services more frequently, which in turn might increase their likelihood of a dementia diagnosis.^26^

Our study showed that the risk of dementia was highest between 3 months and 1 year after an infection. Dementia diagnosed shortly after infection is likely to reflect previously undiagnosed dementia, which is likely to be more prevalent in older adults because the prevalence of dementia increases with age. Systemic inflammation induced by systemic infections has been suggested to accelerate trajectories of cognitive decline in individuals with Alzheimer's disease.^27^, ^28^ Hence, accelerated cognitive decline and dementia progression could have increased the likelihood of a dementia diagnosis. Therefore, our finding of a higher dementia risk shortly after infection supports the need for future studies to clarify whether common infections accelerate cognitive decline and dementia progression in individuals with pre-existing cognitive impairment. Another explanation for this finding could be that older adults presenting with infections in primary care or during hospitalisation who are experiencing cognitive impairment might be referred for cognitive assessment after recovery, which could also increase their chances of receiving a dementia diagnosis.

A common consequence of serious infections is delirium. Delirium itself is a strong risk factor for dementia and can accelerate cognitive decline in individuals with dementia.^29^, ^30^ Delirium can also result in hospitalisation, and, in turn, hospitalisation, particularly in cases requiring intensive care unit admission, has been associated with long-term cognitive impairment.^31^

There are several limitations to our study. First, missed dementia diagnoses were possible because dementia is known to be frequently underdiagnosed in primary care.^32^ However, the number of missed diagnoses has reduced as a result of government policies and strategies, such as the introduction of the Quality and Outcomes Framework in 2004 and the National Dementia Strategy in 2009 and dementia ascertainment in our study was probably improved by using two different data sources.^33^, ^34^, ^35^, ^36^ Misclassification of dementia subtype was probable, particularly in the older age groups in whom dementia is often associated with mixed pathologies.^25^ Furthermore, it is possible that individuals diagnosed with infections had greater health-seeking behaviours; therefore, they were more likely to encounter health services, increasing the likelihood of a dementia diagnosis. This bias would probably be differential and would bias effect estimates away from the null. However, because individuals could be diagnosed with infections at any point during follow-up, we could not explore health-seeking behaviours in people with and without infections before follow-up. Second, ascertainment of cognitive impairment has not been validated in CPRD, and individuals were unlikely to have had their cognitive function tested at multiple timepoints by their general practitioner. However, we excluded individuals with evidence of cognitive impairment before the start of the study, reducing the potential for misclassification. Third, infections are often diagnosed without microbiological data, which increases the possibility of overdiagnosis. To minimise this, individuals were only defined as having urinary tract infections or skin and soft tissue infections if they were treated with antibiotics. Including antibiotic use in the definition of urinary tract infections or skin and soft tissue infections probably increased the specificity of our infection definition; however, defining these infections with antibiotics means that the associations we observed will have incorporated any mitigating effect of antibiotic treatment. As such, the observed associations for these infections might have been underestimated. Asymptomatic infections might also be missed by general practitioners, but these milder infections are less likely to lead to significant systemic inflammation and other pathophysiological mechanisms underlying the association between infections and dementia. Additionally, this bias due to milder infections being missed is probably non-differential by dementia status and would bias effect estimates towards the null. Fourth, adjustment for confounders had a modest effect on our effect estimates, suggesting that our observed association was driven either by residual confounding or a possible causal effect. Although we accounted for a wide range of confounders, which were ascertained with both primary and secondary care data to minimise residual confounding, we cannot rule out the possibility of residual confounding through unmeasured confounders, such as frailty and genetic susceptibility, adjustment for categorical variables, and complex interaction between variables. Frailty is associated with a poor inflammatory response and slower recovery after infections. Therefore, individuals who develop more severe infections might be frailer, which could increase their risk of dementia. Frailty is also associated with adverse outcomes and more comorbidities, some of which we accounted for in our analyses. Finally, due to the long preclinical phase of dementia, we cannot rule out the possibility of reverse causality. People living with dementia are more susceptible to infections and have an increased risk of hospitalisation, with urinary tract infections and pneumonia being two of the most common complications in those hospitalised.^37^ However, to minimise the possibility of reverse causality, we excluded individuals with a history of dementia; furthermore, the association of infections on dementia persisted for more than 9 years, making reverse causality highly unlikely to account for all of the observed effect.

In conclusion, our findings suggest that common infections are associated with an increased risk of dementia in adults (≥65 years), with the risk varying according to type, clinical setting, frequency, and timing of infections, and to the presence of diabetes. Future large-scale, longitudinal studies with a long follow-up period are needed to confirm our findings and to improve our understanding of the mechanisms underlying the associations between infections, diabetes, and dementia. To translate our findings into clinical practice, future studies should investigate whether infection prevention and control interventions reduce the risk of dementia in high-risk populations. Our findings highlight the importance of managing long-term neurological complications following other infections, such as COVID-19, which uniquely has been associated with cognitive dysfunction soon after illness and might also more generally be associated with cognitive decline and dementia in line with the observations made in this study.^38^ Infections, such as periodontitis, for which patients do not typically present to the general practitioner, have been associated with Alzheimer's disease, but these infections were not included in our study; more research is needed in this area.^39^ Additional research is warranted to identify potential microorganisms responsible for the association between infections and dementia. Future studies are also needed to investigate the association between infections and biomarkers related to cognitive impairment and dementia, and to examine the effect of infections on cognitive decline, using validated repeated measures assessing multiple domains of cognition.

## Data sharing

All code lists used in this study and the study protocol online. Data are not publicly available. Access to data is obtained through Clinical Practice Research Datalink and is dependent upon approval of a study protocol by the independent scientific advisory committee.

## Declaration of interests

We declare no competing interests.
