# Supplementary material for: Assessment of common infections and incident dementia using UK primary and secondary care data: a historical cohort study
Source: Lancet Healthy Longev. 2021 Jul;2(7):e426–35. doi: 10.1016/S2666-7568(21)00118-5 (PMC8245326; doi:10.1016/S2666-7568(21)00118-5)
Supplement: Supplementary appendix [file mmc1.pdf]

THE LANCET
Healthy Longevity

## Supplementary appendix

This appendix formed part of the original submission and has been peer reviewed. We post it as supplied by the authors.

Supplement to: Muzambi R, Bhaskaran K, Smeeth L, et al. Assessment of common infections and incident dementia using UK primary and secondary care data: a historical cohort study. *Lancet Healthy Longev* 2021; published online June 18. https://doi.org/10.1016/S2666-7568(21)00118-5.

# Supplementary appendix

## Supplementary Appendix 1: Search strategy

### a) MEDLINE (OVID) search strategy

1. Pneumonia/ or pneumonia, bacterial/
2. Pneumonia.ti,ab
3. Lower respiratory tract infection*.ti,ab
4. (LRTI or LRTIS).ti,ab.
5. Exp urinary tract infections/
6. (Urinary adj5 infection*).ti,ab.
7. (UTI or UTIS).ti,ab
8. exp Cystitis/
9. (bacteriuria or pyuria or cystitis or pyelonephritis or cellulitis).ti,ab.
10. exp cellulitis/
11. (Skin and soft tissue infection).mp.
12. exp sepsis/
13. (septic* or sepsis or septic?emia or systematic inflammatory response syndrome or blood stream infection or py?emia).ti,ab.
14. 1 or 2 or 3 or 4 or 5 or 6 or 7 or 8 or 9 or 10 or 11 or 12 or 13
15. Exp dementia/
16. Exp prion diseases/
17. (huntington* or kluver-bucy or prion disease or Creutzfeldt-jakob or primary progressive aphasia).ti,ab
18. (Dement* or Alzheimer*).ti,ab
19. (Lewy*adj2 bod*).ti,ab.
20. Cognitive dysfunction/
21. (Mild cognitive impairment or MCI).ti,ab
22. ((cognit* or memory or cerebr* or mental*) adj3 (declin* or impair* or los* or deteriorat* or degenerat* or complain* or disturb* or disorder*)).ti,ab.
23. Cognitive function.ti,ab.
24. 13 or 14 or 15 or 16 or 17 or 18 or 19 or 20 or 21 or 22 or 23

25. cohort studies/ or longitudinal study/ or follow-up study/ or prospective study/ or retrospective study/ or cohort.ti,ab. or longitudinal.ti,ab. or prospective.ti,ab. or retrospective.ti,ab.

26. Case-Control Studies/ or Control Groups/ or Matched-Pair Analysis/ or ((case* adj5 control*) or (case adj3 comparison*) or control group*).ti,ab.

27. Incidence/ or incidence.ti,ab,kw.

28. (hazard ratio or HR or odds ratio or relative risk or RR).ti,ab.

29. 25 or 26 or 27 or 28

30. 14 and 24 and 29

**b) Embase (OVID) search strategy**

1. Bacterial pneumonia/ or Pneumonia/
2. Pneumonia.ti,ab
3. Lower respiratory tract infection*.ti,ab
4. (LRTI or LRTIS).ti,ab.
5. Exp Urinary tract infections/
6. exp Bacteriuria/
7. exp Pyuria/
8. (Urinary adj5 infection*).ti,ab.
9. (UTI or UTIS).ti,ab
10. Exp cystitis/
11. (bacteriuria or pyuria or cystitis or pyelonephritis or cellulitis).ti,ab.
12. exp cellulitis/
13. (Skin and soft tissue infection).mp.
14. exp sepsis/
15. (septic* or sepsis or septic?emia or systematic inflammatory response syndrome or blood stream infection or py?emia).ti,ab.
16. 1 or 2 or 3 or 4 or 5 or 6 or 7 or 8 or 9 or 10 or 11 or 12 or 13 or 14 or 15
17. Exp dementia
18. Exp Creutzfeldt-jakob disease
19. (huntington* or kluver-bucy or prion disease or Creutzfeldt-jakob or primary progressive aphasia).ti,ab
20. Dement*.ti,ab.
21. Exp mild cognitive impairment/
22. (Mild cognitive impairment or MCI)ti,ab
23. ((cognit* or memory or cerebr* or mental*) adj3 (declin* or impair* or los* or deteriorat* or degenerat* or complain* or disturb* or disorder*)).ti,ab.
24. Cognitive function.ti,ab.
25. Alzheimer*.ti,ab
26. (Lewy*adj2 bod*).ti,ab.
27. 17 or 18 or 19 or 20 or 21 or 22 or 23 or 24 or 25 or 26

28. cohort analysis/ or longitudinal study/ or follow-up/ or prospective study/ or retrospective studies/ or cohort.ti,ab. or longitudinal.ti,ab. or prospective.ti,ab. or retrospective.ti,ab.

29. Case control study/ or Control Group/ or ((case* adj5 control*) or (case adj3 comparison*) or control group*).ti,ab.

30. Incidence/ or incidence.ti,ab,kw.

31. (hazard ratio or HR or odds ratio or relative risk or RR).ti,ab.

32. 28 or 29 or 30 or 31

33. 16 and 27 and 32

**Supplementary Appendix 2: Directed acyclic graphic depicting the possible confounders and effect modifiers in the association between common infections and dementia.**

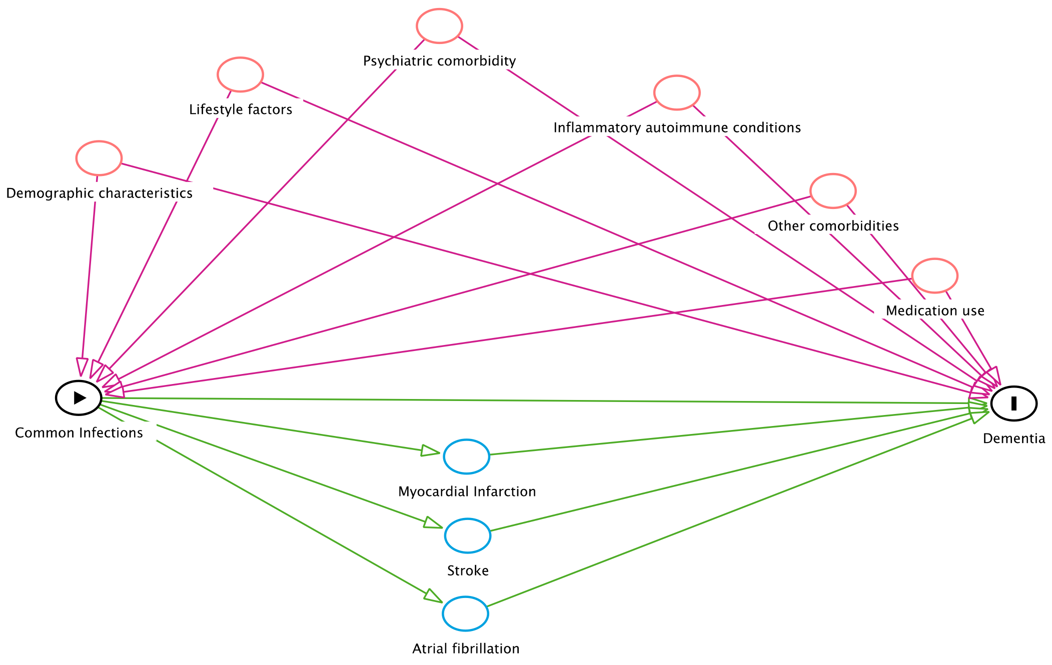


**Legend:**

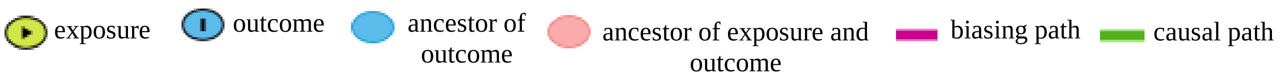

**Supplementary Appendix 3: Variable definitions**

**Infections**

*First ever infection*

Infections were identified using primary care data from the Clinical Practice Research Datalink (CPRD) and secondary care data from Hospital Episode Statistics (HES). In order to confirm a diagnosis of infections and capture more serious infections, urinary tract infections (UTIs) and skin and soft tissue infections (SSTIs) were defined using a Read code and a prescription for antibiotics on the same date as a diagnosis of infection. Sepsis, pneumonia and other lower respiratory tract infections (LRTIs) were not defined using a prescription for antibiotics as these infections can also be caused by viruses. Infections identified in HES were not linked with a prescription for antibiotics as medication data is not available in HES and hospitalised infections are likely to be more serious than those diagnosed in the community.

To avoid misclassification of dementia, we excluded the first 3 months after an infection. That is, when an individual was diagnosed with an infection, they exited the study for 3 months and re-entered after the 3-month exit period.

*Type and clinical setting of infection*

For instances in which an individual was diagnosed with two different types of infections on the same date, a hierarchical approach was used based on the infection type and data source. If two different infections were diagnosed on the same date in CPRD and HES, the infection recorded in HES was used as the primary diagnosis. Then, if sepsis was diagnosed on the same date as another infection, sepsis was the primary diagnosis. If pneumonia and other lower respiratory tract infections were diagnosed on the same date, then the pneumonia diagnosis was taken. If two infections were diagnosed in hospital and GP records, the infection diagnosed in hospital was taken.

*Frequency of infections*

Individuals who were diagnosed with subsequent infections during the 3-month exit period remained out of the study 3 months from subsequent infection diagnosis.

**Covariates**

*Socioeconomic deprivation*

We linked CPRD to patient-level Index of Multiple Deprivation 2015 (IMD 2015). The IMD 2015 is a measure of relative deprivation for small areas across England based on seven domains which can be divided into groups ranking from least to most deprived.(1) Socioeconomic deprivation was defined using patient-level Index of Multiple Deprivation (IMD) which includes only English GP practices. Socioeconomic deprivation was categorised into quintiles; 1 least deprived and 5 most deprived.(1)

*Ethnicity*

Ethnicity was categorised as follows: White, South Asian, Black and Mixed/Other. Using CPRD, we used the most commonly recorded ethnicity, then we used the most recent ethnicity where several ethnicities were recorded. When ethnicity in CPRD was missing, we then used ethnicity recorded in HES. The algorithm we used for ethnicity in CPRD and HES has been previously described.(2)

*BMI and smoking status*

We derived BMI at entry into the study from the CPRD additional details file and defined using methods previously described.(3) We excluded records when an individual was under 16 years, during pregnancy or records under 20kg. BMI was calculated using weight records with height recorded on the same date. However, if a height record on the same date as weight record was not available, we used an older height record. If not available, we used a future height record. The following cut-off points by the World Health Organisation (WHO) were used to define BMI; underweight (<18.5), normal weight (18.5-24.9), overweight (25.0-29.9), obese (30.0>). Smoking status was defined in CPRD, using Read codes and data from the additional details file. Smoking and BMI status were assigned using the nearest record in the period of -1 year to +1 month from start of follow up, if available (best). If not available, the second option was to use the nearest record in the period +1month to +1 year after start of follow up. If not available, the third option was to take the nearest record before -1 year from start of follow up and if not available the least best option was to take any nearest record after +1 year from start of follow up.

*Depression and anxiety*

Depression and anxiety were also defined in CPRD and HES. In CPRD, we included individuals with:

1) a diagnostic or symptomatic Read code for depression or anxiety and

2) a prescription for antidepressants or medications indicated for treatment of anxiety in the British National Formulary (BNF) within 90 days of clinical code.

Depression and anxiety were defined as above due to the changes in diagnosing depression in the UK primary care given that in 2006 GPs switched from using diagnostic to symptomatic codes (4). Additionally, from 2004 antidepressants were no longer routinely prescribed for mild depression, therefore to increase the likelihood of capturing those with more severe depression, we defined depression with a clinical code and prescription for antidepressants (5). Given that depression and anxiety have overlapping clinical codes and medication, we combined the variables for depression and anxiety together. In HES, we used ICD-10 codes alone as individuals diagnosed in hospital are more likely to be severe than those in diagnosed in primary care.

*Diabetes*

We defined diabetes using diagnoses for diabetes mellitus (type 1, type 2 and unspecified) and codes for diabetes complications. To define diabetes, we used Read codes in CPRD and ICD-10 codes in HES. Codes for gestational diabetes, secondary diabetes such as "diabetes mellitus induced by steroids" and diabetes care codes were excluded.

*Polypharmacy*

We defined polypharmacy as the concurrent use of 5 or more medications using BNF chapters. We excluded vaccines and devices that do not administer medication. Medication use was captured in the 12 months prior to baseline.

**REFERENCES**

1. Department for Communities and Local Government. English indices of deprivation 2015 : Department for Communities and Local Government,; 2016 [cited Department for Communities and Local Government, . Available from: https://assets.publishing.service.gov.uk/government/uploads/system/uploads/attachment_data/file/579151/English_Indices_of_Deprivation_2015_-_Frequently_Asked_Questions_Dec_2016.pdf.
2. Mathur R, Bhaskaran K, Chaturvedi N, Leon DA, vanStaa T, Grundy E, et al. Completeness and usability of ethnicity data in UK-based primary care and hospital databases. J Public Health (Oxf). 2014;36(4):684-92.
3. Bhaskaran K, Forbes HJ, Douglas I, Leon DA, Smeeth L. Representativeness and optimal use of body mass index (BMI) in the UK Clinical Practice Research Datalink (CPRD). BMJ Open. 2013;3(9):e003389.
4. Kendrick T, Stuart B, Newell C, Geraghty AW, Moore M. Changes in rates of recorded depression in English primary care 2003-2013: Time trend analyses of effects of the economic recession, and the GP contract quality outcomes framework (QOF). J Affect Disord. 2015;180:68-78.
5. Carreira H, Williams R, Strongman H, Bhaskaran K. Identification of mental health and quality of life outcomes in primary care databases in the UK: a systematic review. BMJ Open. 2019;9(7):e029227.

**Supplementary Table 1: Sensitivity analyses**

| Sensitivity analysis | Justification |
|---|---|
| Primary analyses repeated excluding individuals with secondary dementia causally related to other conditions. | Infections are unlikely to be causally associated with this type of dementia |
| Primary analyses repeated defining all infections in CPRD with a diagnostic code and prescription for antibiotics | To improve the accuracy of our infection definition |
| Primary analysis repeated excluding individuals diagnosed with infections from two different sites (e.g. skin and soft tissue infections and pneumonia) on the same date | To avoid any potential biases introduced from including these infections. |
| We excluded codes relating to symptoms of cognitive impairment from analysis of infections and cognitive impairment | To improve the accuracy of our definition of cognitive impairment |

**Supplementary Figures**

**Supplementary Figure 1: Flowchart of study population**

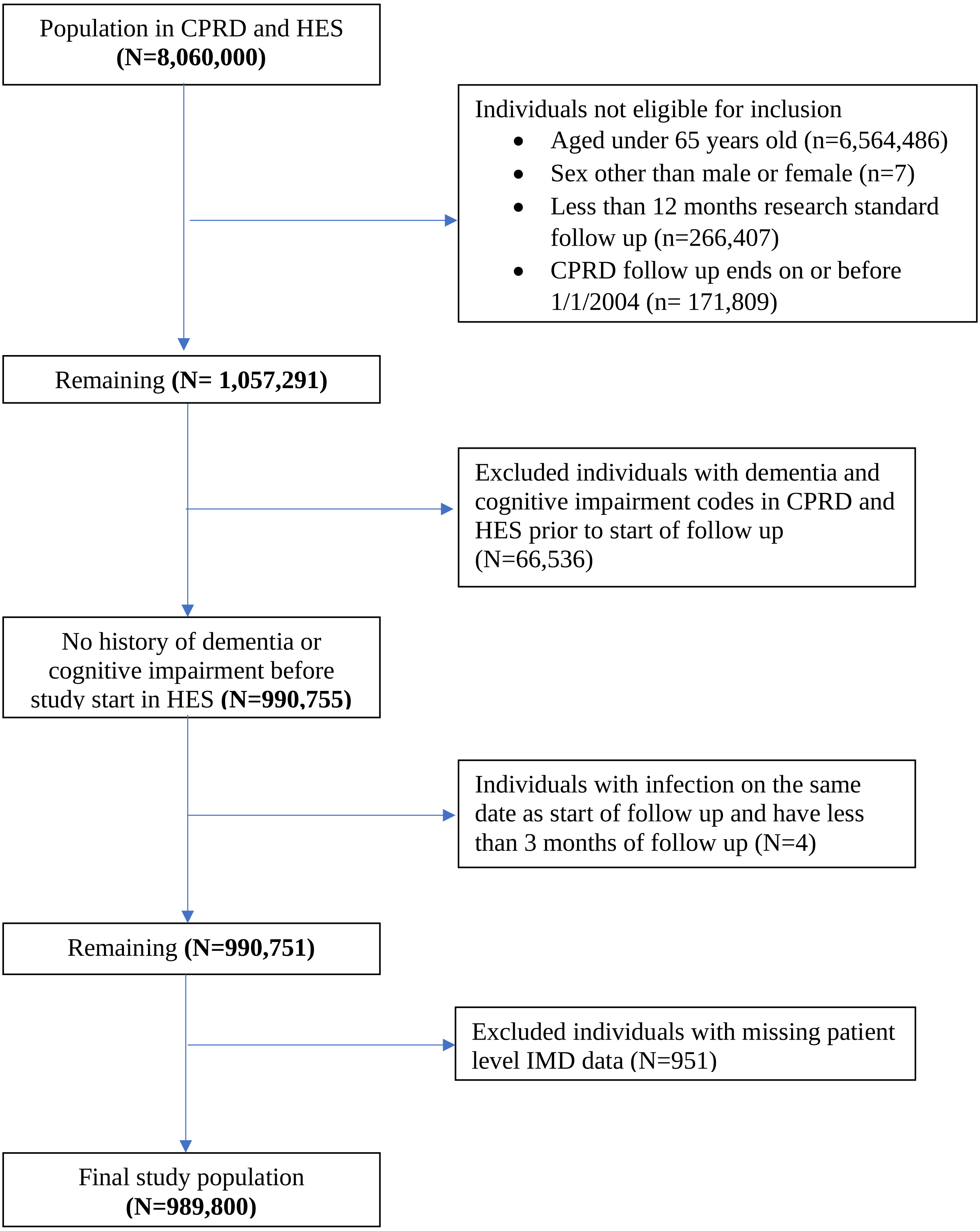

**Supplementary Table 2. Age-specific incidence rates of dementia during person-time with and without prior common infections.**

| Age group (years) | Person-years | Total number of incident dementia diagnoses | Rate (per 1000-person years) | Lower (95% CI) | Upper (95% CI) |
|---|---|---|---|---|---|
| **No infections** | | | | | |
| 65-69 | 1395965 | 1263 | 0.90 | 0.86 | 0.96 |
| 70-74 | 935486 | 2523 | 2.70 | 2.59 | 2.80 |
| 75-79 | 693243 | 4684 | 6.76 | 6.57 | 6.95 |
| 80-84 | 481002 | 6697 | 13.92 | 13.59 | 14.26 |
| 85-89 | 258807 | 6079 | 23.49 | 22.91 | 24.09 |
| 90+ | 130528 | 4068 | 31.17 | 30.22 | 32.14 |
| Total | 3895032 | 25314 | 6.50 | 6.42 | 6.58 |
| **Any infections** | | | | | |
| 65-69 | 260196 | 544 | 2.09 | 1.92 | 2.27 |
| 70-74 | 426037 | 2104 | 4.94 | 4.73 | 5.15 |
| 75-79 | 408733 | 4681 | 11.45 | 11.13 | 11.79 |
| 80-84 | 331452 | 7749 | 23.38 | 22.86 | 23.91 |
| 85-89 | 212080 | 8865 | 41.80 | 40.94 | 42.68 |
| 90+ | 116458 | 7545 | 64.79 | 63.34 | 66.27 |
| Total | 1754956 | 31488 | 17.94 | 17.75 | 18.14 |

**Supplementary Table 3. Crude rate and hazard ratios for the association of common infections and dementia, additionally adjusted for potential mediators and BMI**

| Infection | Total number of incident dementia diagnoses | Person-years at risk | Crude incidence rate (95% CI) | *Fully adjusted HR | **Additionally adjusted for potential mediators HR | ***Additionally adjusted for BMI HR |
|---|---|---|---|---|---|---|
| No infection | 25314 | 3895032 | 6.50 (6.42-6.58) | 1.00 | 1.00 | 1.00 |
| Any Infection | 31488 | 1754956 | 17.94 (17.75-18.14) | 1.53 (1.50-1.55) | 1.65 (1.62-1.68) | 1.53 (1.50-1.56) |
| Sepsis | 427 | 16814 | 25.40 (23.10-27.92) | 2.08 (1.89-2.29) | 2.32 (2.11-2.56) | 2.07 (1.87-2.29) |
| Pneumonia | 1247 | 47836 | 26.07 (24.66-27.56) | 1.88 (1.77-1.99) | 2.11 (2.00-2.24) | 1.88 (1.77-2.00) |
| Other LRTI | 13429 | 910432 | 14.75 (14.50-15.00) | 1.34 (1.31-1.37) | 1.46 (1.42-1.49) | 1.35 (1.32-1.38) |
| UTI | 10513 | 481341 | 21.84 (21.43-22.26) | 1.73 (1.69-1.78) | 1.86 (1.82-1.91) | 1.73 (1.68-1.77) |
| SSTI | 5535 | 291603 | 18.98 (18.49-19.49) | 1.54 (1.49-1.58) | 1.67 (1.62-1.72) | 1.57 (1.52-1.62) |

HR, hazard ratio; LRTIs, lower respiratory tract infections (excluding pneumonia); UTI; urinary tract infection, SSTIs, skin and soft tissue infection
*Age as underlying time scale. * Adjusted for age, sex, patient level IMD, calendar period, ethnicity, smoking status, heavy alcohol consumption, anxiety and depression, severe mental illness, inflammatory bowel disease, multiple sclerosis, rheumatoid arthritis, psoriasis, asthma, chronic kidney disease, chronic liver disease, chronic obstructive pulmonary disease, diabetes mellitus, heart failure, hypertension, obstructive sleep apnoea, stroke, traumatic brain injury, benzodiazepines, proton pump inhibitors, systemic corticosteroids and polypharmacy. **Additionally adjusted for atrial fibrillation, myocardial infarction and stroke. ***Fully adjusted model additionally adjusted for BMI

**Supplementary Figure 2. Forest plot depicting adjusted hazard ratios of the association between common infections and dementia, stratified according to time since infection (non-overlapping time periods)**

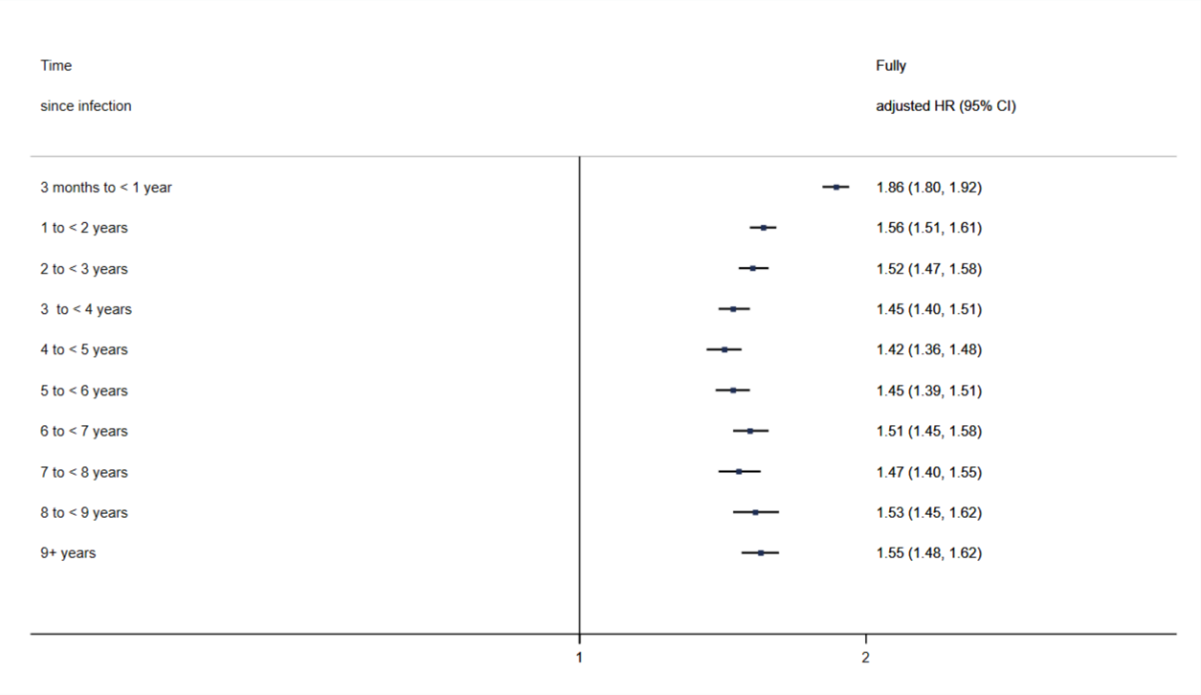


HR, hazard ratio. Adjusted for age, sex, patient level IMD, calendar period, ethnicity, smoking status, heavy alcohol consumption, anxiety and depression, severe mental illness, inflammatory bowel disease, multiple sclerosis, rheumatoid arthritis, psoriasis, asthma, chronic kidney disease, chronic liver disease, chronic obstructive pulmonary disease, diabetes mellitus, heart failure, hypertension, obstructive sleep apnoea, stroke, traumatic brain injury, benzodiazepines, proton pump inhibitors, systemic corticosteroids and polypharmacy.

**Supplementary Table 4. Crude rate and hazard ratios for the association of common infections and dementia in people with and without diabetes**

| Infection | Total number of incident dementia diagnoses | Total person-years at risk | Crude incidence rate (95% CI) | *Age-adjusted HR (95% CI) | **Age, sex, IMD and calendar period adjusted HR (95% CI) | ***Fully adjusted HR (95% CI) |
|---|---|---|---|---|---|---|
| **Individuals without diabetes** | | | | | | |
| No infection | 22610 | 3523440 | 6·42 (6·33-6·50) | 1·00 | 1·00 | 1·00 |
| Any Infection | 27141 | 1543709 | 17·58 (17·37-17·79) | 1·75 (1·72-1·78) | 1·61 (1·58-1·64) | 1·50 (1·47-1·53) |
| **Individuals with diabetes** | | | | | | |
| No infection | 2704 | 371592 | 7·28 (7·01-7·56) | 1·00 | 1·00 | 1·00 |
| Any Infection | 4347 | 211247 | 20·58 (19·98-21·20) | 1·94 (1·85-2·04) | 1·74 (1·66-1·83) | 1·70 (1·61-1·79) |

HR, hazard ratio; Age as the underlying time scale. ** Adjusted for age, sex, ethnicity, patient level IMD and calendar period *** additionally adjusted for ethnicity, smoking status, heavy alcohol consumption, anxiety and depression, severe mental illness, inflammatory bowel disease, multiple sclerosis, rheumatoid arthritis, psoriasis, asthma, chronic kidney disease, chronic liver disease, chronic obstructive pulmonary disease, heart failure, hypertension, obstructive sleep apnoea, stroke, traumatic brain injury, benzodiazepines, proton pump inhibitors, systemic corticosteroids and polypharmacy. Likelihood ratio test for interaction comparing models with and without interaction term between infections and diabetes, p=0.00014.

**Supplementary Table 5. Crude rate and hazard ratios for the association of common infections and cognitive impairment, stratified by type of infection**

| Infection | Total number of incident cognitive impairment events | Total person-years at risk | Crude incidence rate (95% CI) | *Age-adjusted HR (95% CI) | **Age, sex, IMD and calendar period adjusted HR (95% CI) | ***Fully adjusted HR (95% CI) |
|---|---|---|---|---|---|---|
| No infection | 34730 | 3855389 | 9·01 (8·91-9·10) | 1·00 | 1·00 | 1·00 |
| Any Infection | 32608 | 1679805 | 19·41 (19·20-19·62) | 1·57 (1·55-1·60) | 1·45 (1·42-1·47) | 1·29 (1·27-1·32) |
| Sepsis | 340 | 15763 | 21·57 (19·39-23·99) | 1·74 (1·56-1·93) | 1·57 (1·41-1·75) | 1·39 (1·25-1·55) |
| Pneumonia | 1110 | 44972 | 24·68 (23·27-26·18) | 1·83 (1·72-1·94) | 1·65 (1·56-1·75) | 1·45 (1·36-1·54) |
| Other LRTI | 15527 | 875906 | 17·73 (17·45-18·01) | 1·50 (1·47-1·53) | 1·38 (1·35-1·41) | 1·22 (1·20-1·25) |
| UTI | 9627 | 457755 | 21·03 (20·61-21·46) | 1·63 (1·60-1·67) | 1·51 (1·48-1·55) | 1·36 (1·33-1·40) |
| SSTI | 5787 | 279042 | 20·74 (20·21-21·28) | 1·61 (1·56-1·65) | 1·48 (1·43-1·52) | 1·34 (1·30-1·38) |

HR, hazard ratio; LRTIs, lower respiratory tract infections (excluding pneumonia); UTI; urinary tract infection, SSTI, skin and soft tissue infection
*Age as the underlying time scale. ** Adjusted for age, sex, patient level IMD and calendar period *** additionally adjusted for ethnicity, smoking status, heavy alcohol consumption, anxiety and depression, severe mental illness, inflammatory bowel disease, multiple sclerosis, rheumatoid arthritis, psoriasis, asthma, chronic kidney disease, chronic liver disease, chronic obstructive pulmonary disease, diabetes mellitus, heart failure, hypertension, obstructive sleep apnoea, stroke, traumatic brain injury, benzodiazepines, proton pump inhibitors, systemic corticosteroids and polypharmacy.

**Supplementary Table 6: Crude rate and hazard ratios for the association of common infections and dementia, stratified by dementia subtype**

| Infection | Total number of incident dementia diagnoses | Person-years at risk | Crude incidence rate (95% CI) | *Age-adjusted HR (95% CI) | **Age, sex, IMD and calendar period adjusted HR (95% CI) | ***Fully adjusted HR (95% CI) |
|---|---|---|---|---|---|---|
| **Alzheimer's Disease** | | | | | | |
| No infection | 7137 | 3895032 | 1.83 (1.79-1.88) | 1.00 | 1.00 | 1.00 |
| Any Infection | 6424 | 1754956 | 3.66 (3.57-3.75) | 1.33 (1.29-1.38) | 1.12 (1.08-1.16) | 1.09 (1.05-1.13) |
| Sepsis | 54 | 16814 | 3.21 (2.46-4.19) | 1.16 (0.89-1.52) | 0.99 (0.75-1.29) | 0.98 (0.75-1.28) |
| Pneumonia | 203 | 47836 | 4.24 (3.70-4.87) | 1.40 (1.21-1.61) | 1.19 (1.04-1.37) | 1.15 (1.00-1.33) |
| Other LRTI | 2956 | 910432 | 3.25 (3.13-3.37) | 1.25 (1.20-1.30) | 1.07 (1.02-1.12) | 1.03 (0.99-1.08) |
| UTI | 2050 | 481341 | 4.26 (4.08-4.45) | 1.47 (1.40-1.55) | 1.21 (1.15-1.27) | 1.17 (1.11-1.23) |
| SSTI | 1126 | 291603 | 3.86 (3.64-4.09) | 1.34 (1.26-1.43) | 1.13 (1.06-1.21) | 1.09 (1.02-1.17) |
| **Vascular Dementia** | | | | | | |
| No infection | 5040 | 3895032 | 1.29 (1.26-1.33) | 1.00 | 1.00 | 1.00 |
| Any Infection | 7132 | 1754956 | 4.06 (3.97-4.16) | 2.06 (1.98-2.13) | 1.88 (1.81-1.95) | 1.69 (1.62-1.76) |
| Sepsis | 119 | 16814 | 7.08 (5.91-8.47) | 3.54 (2.96-4.25) | 3.16 (2.63-3.79) | 2.74 (2.28-3.29) |
| Pneumonia | 295 | 47836 | 6.17 (5.50-6.91) | 2.79 (2.48-3.13) | 2.46 (2.19-2.77) | 2.08 (1.85-2.35) |
| Other LRTI | 3136 | 910432 | 3.44 (3.33-3.57) | 1.85 (1.77-1.94) | 1.68 (1.60-1.75) | 1.50 (1.43-1.58) |
| UTI | 2262 | 481341 | 4.70 (4.51-4.90) | 2.24 (2.14-2.36) | 2.11 (2.01-2.22) | 1.89 (1.79-1.99) |
| SSTI | 1242 | 291603 | 4.26 (4.03-4.50) | 2.05 (1.92-2.18) | 1.87 (1.76-1.99) | 1.69 (1.59-1.80) |
| **Unspecified Dementia** | | | | | | |
| No infection | 12210 | 3895032 | 3.13 (3.08-3.19) | 1.00 | 1.00 | 1.00 |
| Any Infection | 16956 | 1754956 | 9.66 (9.52-9.81) | 1.92 (1.88-1.97) | 1.84 (1.80-1.89) | 1.72 (1.67-1.76) |
| Sepsis | 247 | 16814 | 14.69 (12.97-16.64) | 2.86 (2.52-3.24) | 2.79 (2.46-3.17) | 2.51 (2.21-2.85) |
| Pneumonia | 716 | 47836 | 14.97 (13.91-16.11) | 2.55 (2.36-2.75) | 2.46 (2.28-2.65) | 2.22 (2.05-2.39) |
| Other LRTI | 6909 | 910432 | 7.59 (7.41-7.77) | 1.64 (1.59-1.69) | 1.57 (1.53-1.62) | 1.46 (1.41-1.51) |
| UTI | 5861 | 481341 | 12.18 (11.87-12.49) | 2.25 (2.18-2.33) | 2.14 (2.07-2.21) | 1.99 (1.92-2.05) |
| SSTI | 3009 | 291603 | 10.32(9.96-10.69) | 1.93 (1.85-2.00) | 1.86 (1.79-1.94) | 1.74 (1.67-1.81) |

HR, hazard ratio; LRTIs, lower respiratory tract infections (excluding pneumonia); UTI; urinary tract infection, SSTIs, skin and soft tissue infection
*Age as underlying time scale. ** Adjusted for age, sex, patient level IMD and calendar period *** additionally adjusted for ethnicity, smoking status, heavy alcohol consumption, anxiety and depression, severe mental illness, inflammatory bowel disease, multiple sclerosis, rheumatoid arthritis, psoriasis, asthma, chronic kidney disease, chronic liver disease, chronic obstructive pulmonary disease, diabetes mellitus, heart failure, hypertension, obstructive sleep apnoea, stroke, traumatic brain injury, benzodiazepines, proton pump inhibitors, systemic corticosteroids and polypharmacy

**Supplementary Table 7: Crude rate and hazard ratios for the association of common infections and dementia, stratified by sex**

| Infection | Total number of incident dementia diagnoses | Person-years at risk | Crude incidence rate (95% CI) | *Age-adjusted HR (95% CI) | **Age, sex, IMD and calendar period adjusted HR (95% CI) | ***Fully adjusted HR (95% CI) |
|---|---|---|---|---|---|---|
| **Female** | | | | | | |
| No infection | 15427 | 2043935 | 7.55 (7.43-7.67) | 1.00 | 1.00 | 1.00 |
| Any infection | 20658 | 1065696 | 19.38 (19.12-19.65) | 1.75 (1.71-1.79) | 1.61 (1.58-1.65) | 1.51 (1.48-1.55) |
| Sepsis | 219 | 7641 | 28.66 (25.11-32.72) | 2.51 (2.20-2.87) | 2.30 (2.01-2.63) | 2.11 (1.84-2.42) |
| Pneumonia | 650 | 21756 | 29.88 (27.67-32.26) | 2.20 (2.03-2.38) | 1.99 (1.84-2.16) | 1.81 (1.67-1.96) |
| Other LRTI | 7979 | 495893 | 16.09 (15.74-16.45) | 1.55 (1.51-1.59) | 1.42 (1.38-1.46) | 1.33 (1.29-1.37) |
| UTI | 8124 | 373232 | 21.77 (21.30-22.25) | 1.94 (1.89-1.99) | 1.80 (1.75-1.85) | 1.68 (1.64-1.73) |
| SSTI | 3469 | 162895 | 21.30 (20.60-22.02) | 1.73 (1.66-1.79) | 1.59 (1.53-1.65) | 1.51 (1.45-1.57) |
| **Male** | | | | | | |
| No infection | 9887 | 1851097 | 5.34 (5.24-5.45) | 1.00 | 1.00 | 1.00 |
| Any infection | 10830 | 689261 | 15.71 (15.42-16.01) | 1.83 (1.78-1.88) | 1.69 (1.64-1.74) | 1.56 (1.51-1.60) |
| Sepsis | 208 | 9173 | 22.67 (19.79-25.98) | 2.53 (2.21-2.91) | 2.30 (2.01-2.64) | 2.08 (1.81-2.39) |
| Pneumonia | 597 | 26080 | 22.89 (21.13-24.80) | 2.42 (2.23-2.63) | 2.20 (2.03-2.39) | 1.98 (1.82-2.15) |
| Other LRTI | 5450 | 414539 | 13.15 (12.80-13.50) | 1.61 (1.56-1.67) | 1.49 (1.44-1.54) | 1.37 (1.33-1.42) |
| UTI | 2389 | 108109 | 22.10 (21.23-23.00) | 2.25 (2.15-2.36) | 2.09 (2.00-2.19) | 1.90 (1.81-1.99) |
| SSTI | 2066 | 128708 | 16.05 (15.37-16.76) | 1.88 (1.79-1.97) | 1.73 (1.64-1.81) | 1.58 (1.51-1.66) |

HR, hazard ratio; LRTIs, lower respiratory tract infections (excluding pneumonia); UTI; urinary tract infection, SSTIs, skin and soft tissue infection
*Age as underlying time scale. ** Adjusted for age, sex, patient level IMD and calendar period *** additionally adjusted for ethnicity, smoking status, heavy alcohol consumption, anxiety and depression, severe mental illness, inflammatory bowel disease, multiple sclerosis, rheumatoid arthritis, psoriasis, asthma, chronic kidney disease, chronic liver disease, chronic obstructive pulmonary disease, diabetes mellitus, heart failure, hypertension, obstructive sleep apnoea, stroke, traumatic brain injury, benzodiazepines, proton pump inhibitors, systemic corticosteroids and polypharmacy.

**Supplementary Figure 3: Kaplan-Meier survival curve depicting the time to death after a dementia diagnosis in years**

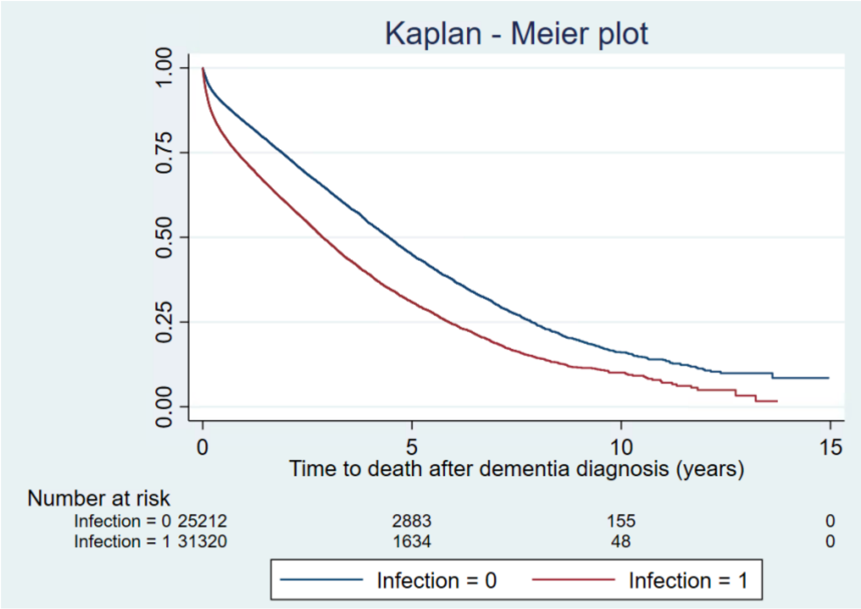


**Supplementary Figure 4: Kaplan-Meier survival curve depicting the time to death 12 months after dementia diagnosis**

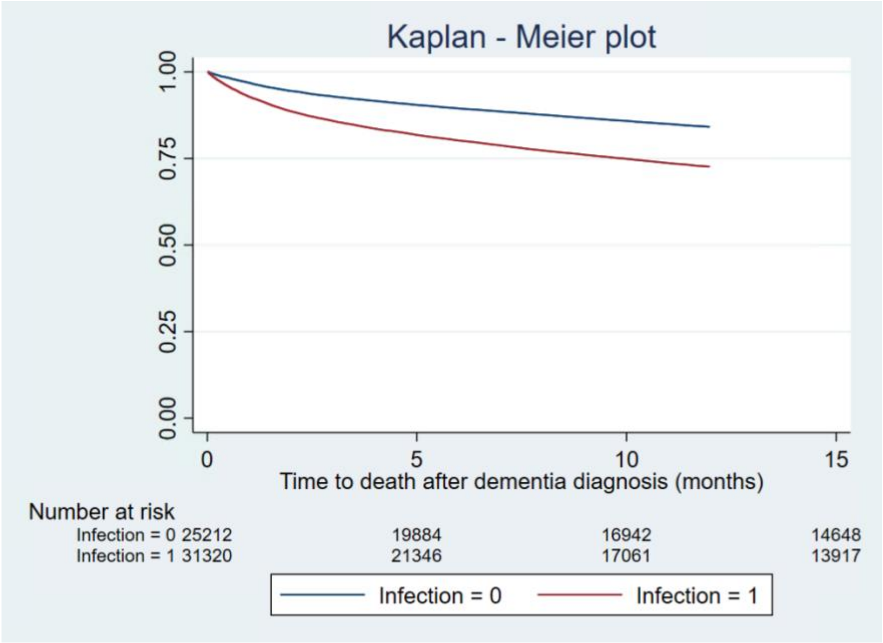

**Supplementary Table 8: Crude rate and hazard ratios for the association of common infections and dementia, stratified by age**

| Infection | Total number of incident dementia diagnoses | Total person-years at risk | Crude incidence rate (95% CI) | *Age-adjusted HR (95% CI) | **Age, sex, IMD and calendar period adjusted HR (95% CI) | ***Fully adjusted HR (95% CI) |
|---|---|---|---|---|---|---|
| **65-79** | | | | | | |
| No infection | 8470 | 3024694 | 2.80 (2.74-2.86) | 1.00 | 1.00 | 1.00 |
| Any Infection | 7329 | 1094966 | 6.69 (6.54-6.85) | 1.72 (1.67-1.78) | 1.60 (1.55-1.66) | 1.43 (1.38-1.48) |
| **80-89** | | | | | | |
| No infection | 12776 | 739810 | 17.27 (16.97-17.57) | 1.00 | 1.00 | 1.00 |
| Any infection | 16614 | 543532 | 30.57 (30.11-31.04) | 1.72 (1.68-1.76) | 1.57 (1.53-1.60) | 1.49 (1.46-1.53) |
| **90 years and older** | | | | | | |
| No infection | 4068 | 130528 | 31.17 (30.22-32.14) | 1.00 | 1.00 | 1.00 |
| Any infection | 7545 | 116458 | 64.79 (63.34-66.27) | 2.07 (1.99-2.15) | 1.89 (1.82-1.97) | 1.76 (1.69-1.84) |

*Age as underlying time scale. ** Adjusted for age, sex, patient level IMD and calendar period *** additionally adjusted for ethnicity, smoking status, heavy alcohol consumption, anxiety and depression, severe mental illness, inflammatory bowel disease, multiple sclerosis, rheumatoid arthritis, psoriasis, asthma, chronic kidney disease, chronic liver disease, chronic obstructive pulmonary disease, diabetes mellitus, heart failure, hypertension, obstructive sleep apnoea, stroke, traumatic brain injury, benzodiazepines, proton pump inhibitors, systemic corticosteroids and polypharmacy.

**Supplementary Table 9: Crude rate and hazard ratios for the association of common infections and dementia, stratified by age (<90 and 90> years)**

| Infection | Total number of incident dementia diagnoses | Total person-years at risk | Crude incidence rate (95% CI) | *Age-adjusted HR (95% CI) | **Age, sex, IMD and calendar period adjusted HR (95% CI) | ***Fully adjusted HR (95% CI) |
|---|---|---|---|---|---|---|
| **<90 years old** | | | | | | |
| No infection | 21246 | 3764504 | 5.64 (5.57-5.72) | 1.00 | 1.00 | 1.00 |
| Any Infection | 23943 | 1638498 | 14.61 (14.43-14.80) | 1.72 (1.69-1.75) | 1.58 (1.55-1.61) | 1.47 (1.44-1.50) |
| **90 years and older** | | | | | | |
| No infection | 4068 | 130528 | 31.17 (30.22-32.14) | 1.00 | 1.00 | 1.00 |
| Any infection | 7545 | 116458 | 64.79 (63.34-66.27) | 2.07 (1.99-2.15) | 1.89 (1.82-1.97) | 1.76 (1.69-1.84) |

*Age as underlying time scale. ** Adjusted for age, sex, patient level IMD and calendar period *** additionally adjusted for ethnicity, smoking status, heavy alcohol consumption, anxiety and depression, severe mental illness, inflammatory bowel disease, multiple sclerosis, rheumatoid arthritis, psoriasis, asthma, chronic kidney disease, chronic liver disease, chronic obstructive pulmonary disease, diabetes mellitus, heart failure, hypertension, obstructive sleep apnoea, stroke, traumatic brain injury, benzodiazepines, proton pump inhibitors, systemic corticosteroids and polypharmacy.

**Supplementary Table 10: Crude rate and hazard ratios for the association of common infections and dementia, with interaction between age and non-proportional covariates** (sex, ethnicity, patient-level IMD, smoking status, heavy alcohol consumption, anxiety and depression, severe mental illness, chronic kidney disease, hypertension, traumatic brain injury, benzodiazepines and polypharmacy)

| Infection | Total number of incident dementia diagnoses | Total person-years at risk | Crude incidence rate (95% CI) | *Age-adjusted HR (95% CI) | **Age, sex, IMD and calendar period adjusted HR (95% CI) | ***Fully adjusted HR (95% CI) |
|---|---|---|---|---|---|---|
| No infection | 25314 | 3895032 | 6.50 (6.42-6.58) | 1.00 | 1.00 | 1.00 |
| Any Infection | 31488 | 1754956 | 17.94 (17.75-18.14) | 1.78 (1.75-1.81) | 1.64 (1.61-1.66) | 1.52 (1.49-1.55) |

*Age as underlying time scale. ** Adjusted for age, sex, patient level IMD and calendar period *** additionally adjusted for ethnicity, smoking status, heavy alcohol consumption, anxiety and depression, severe mental illness, inflammatory bowel disease, multiple sclerosis, rheumatoid arthritis, psoriasis, asthma, chronic kidney disease, chronic liver disease, chronic obstructive pulmonary disease , diabetes mellitus, heart failure, hypertension, obstructive sleep apnoea, stroke, traumatic brain injury, benzodiazepines, proton pump inhibitors, systemic corticosteroids and polypharmacy.

**Supplementary Table 11: Crude rate and hazard ratios for the association of common infections and dementia, excluding dementia related to other diseases**

| Infection | Total number of incident dementia diagnoses | Person-years at risk | Crude incidence rate (95% CI) | *Age-adjusted HR (95% CI) | **Age, sex, IMD and calendar period adjusted HR (95% CI) | ***Fully adjusted HR (95% CI) |
|---|---|---|---|---|---|---|
| No infection | 24387 | 3890864 | 6.27 (6.19-6.35) | 1.00 | 1.00 | 1.00 |
| Any Infection | 30512 | 1751183 | 17.42 (17.23-17.62) | 1.78 (1.75-1.81) | 1.63 (1.61-1.66) | 1.52 (1.50-1.55) |
| Sepsis | 420 | 16789 | 25.02 (22.74-27.53) | 2.52 (2.29-2.78) | 2.32 (2.10-2.55) | 2.11 (1.91-2.32) |
| Pneumonia | 1214 | 47753 | 25.42 (24.03-26.89) | 2.28 (2.15-2.41) | 2.08 (1.96-2.20) | 1.87 (1.77-1.99) |
| Other LRTI | 13001 | 908633 | 14.31 (14.06-14.56) | 1.57 (1.54-1.60) | 1.44 (1.41-1.47) | 1.34 (1.31-1.37) |
| UTI | 10173 | 480182 | 21.19 (20.78-21.60) | 2.03 (1.99-2.08) | 1.85 (1.81-1.90) | 1.71 (1.67-1.76) |
| SSTI | 5377 | 290917 | 18.48 (18.00-18.98) | 1.78 (1.73-1.84) | 1.64 (1.59-1.69) | 1.53 (1.48-1.58) |

HR, hazard ratio; LRTIs, lower respiratory tract infections (excluding pneumonia); UTI; urinary tract infection, SSTIs, skin and soft tissue infection
*Age as underlying time scale. ** Adjusted for age, sex, patient level IMD and calendar period *** additionally adjusted for ethnicity, smoking status, heavy alcohol consumption, anxiety and depression, severe mental illness, inflammatory bowel disease, multiple sclerosis, rheumatoid arthritis, psoriasis, asthma, chronic kidney disease, chronic liver disease, chronic obstructive pulmonary disease, diabetes mellitus, heart failure, hypertension, obstructive sleep apnoea, stroke, traumatic brain injury, benzodiazepines, proton pump inhibitors, systemic corticosteroids and polypharmacy.

**Supplementary Table 12: Crude rate and hazard ratios for the association of common infections and dementia, according to definition of infections**

| Infection | Total number of incident dementia diagnoses | Person-years at risk | Crude incidence rate (95% CI) | *Age-adjusted HR (95% CI) | **Age, sex, IMD and calendar period adjusted HR (95% CI) | ***Fully adjusted HR (95% CI) |
|---|---|---|---|---|---|---|
| **All infections in CPRD treated with antibiotics** | | | | | | |
| No infection | 27548 | 4003253 | 6.88 (6.80-6.96) | 1.00 | 1.00 | 1.00 |
| Any infection | 29503 | 1653011 | 17.85 (17.65-18.05) | 1.71 (1.69-1.74) | 1.57 (1.55-1.60) | 1.47 (1.44-1.50) |
| Sepsis | 372 | 13620 | 27.31 (24.67-30.23) | 2.53 (2.28-2.80) | 2.32 (2.09-2.57) | 2.13 (1.92-2.36) |
| Pneumonia | 1109 | 41212 | 26.91 (25.37-28.54) | 2.23 (2.10-2.37) | 2.03 (1.91-2.16) | 1.84 (1.73-1.95) |
| Other LRTIs | 11635 | 818240 | 14.22 (13.96-14.48) | 1.48 (1.45-1.52) | 1.36 (1.33-1.39) | 1.27 (1.24-1.30) |
| UTI | 10511 | 481330 | 21.84 (21.42-22.26) | 1.96 (1.91-2.00) | 1.79 (1.75-1.84) | 1.67 (1.63-1.71) |
| SSTI | 5535 | 291627 | 18.98 (18.49-19.49) | 1.71 (1.66-1.76) | 1.58 (1.53-1.63) | 1.48 (1.44-1.52) |
| **Excluding multiple infection diagnoses** | | | | | | |
| No infection | 25314 | 3875552 | 6.53 (6.45-6.61) | 1.00 | 1.00 | 1.00 |
| Any infection | 31151 | 1748025 | 17.82 (17.62-18.02) | 1.75 (1.72-1.78) | 1.61 (1.58-1.64) | 1.50 (1.47-1.53) |

HR, hazard ratio; LRTIs, lower respiratory tract infections (excluding pneumonia); UTI; urinary tract infection, SSTIs, skin and soft tissue infection
*Age as underlying time scale. ** Adjusted for age, sex, patient level IMD and calendar period *** additionally adjusted for ethnicity, smoking status, heavy alcohol consumption, anxiety and depression, severe mental illness, inflammatory bowel disease, multiple sclerosis, rheumatoid arthritis, psoriasis, asthma, chronic kidney disease, chronic liver disease, chronic obstructive pulmonary disease, diabetes mellitus, heart failure, hypertension, obstructive sleep apnoea, stroke, traumatic brain injury, benzodiazepines, proton pump inhibitors, systemic corticosteroids and polypharmacy.

**Supplementary Table 13: Crude rate and hazard ratios for the association of common infections and cognitive impairment, excluding codes related to symptoms of cognitive impairment**

| Infection | Total number of incident cognitive impairment diagnoses | Person-years at risk | Crude incidence rate (95% CI) | *Age-adjusted HR (95% CI) | **Age, sex, IMD and calendar period adjusted HR (95% CI) | ***Fully adjusted HR (95% CI) |
|---|---|---|---|---|---|---|
| No infection | 5297 | 3102598 | 1.71 (1.66-1.75) | 1.00 | 1.00 | 1.00 |
| Any Infection | 7243 | 1471353 | 4.92 (4.81-5.04) | 1.93 (1.86-2.00) | 1.74 (1.68-1.81) | 1.62 (1.56-1.68) |
| Sepsis | 97 | 14809 | 6.55 (5.37-7.99) | 2.40 (1.96-2.93) | 2.04 (1.67-2.50) | 1.86 (1.52-2.27) |
| Pneumonia | 316 | 41986 | 7.53 (6.74-8.40) | 2.43 (2.17-2.72) | 2.08 (1.85-2.33) | 1.87 (1.67-2.10) |
| Other LRTI | 3191 | 821156 | 3.89 (3.75-4.02) | 1.50 (1.44-1.57) | 1.33 (1.27-1.39) | 1.21 (1.16-1.27) |
| UTI | 2240 | 425612 | 5.26 (5.05-5.49) | 1.82 (1.73-1.91) | 1.68 (1.60-1.77) | 1.57 (1.49-1.65) |
| SSTI | 1332 | 258961 | 5.14 (4.87-5.43) | 1.78 (1.68-1.89) | 1.58 (1.49-1.68) | 1.48 (1.40-1.58) |

HR, hazard ratio; LRTIs, lower respiratory tract infections (excluding pneumonia); UTI; urinary tract infection, SSTIs, skin and soft tissue infection
*Age as underlying time scale. ** Adjusted for age, sex, patient level IMD and calendar period *** additionally adjusted for ethnicity, smoking status, heavy alcohol consumption, anxiety and depression, severe mental illness, inflammatory bowel disease, multiple sclerosis, rheumatoid arthritis, psoriasis, asthma, chronic kidney disease, chronic liver disease, chronic obstructive pulmonary disease, diabetes mellitus, heart failure, hypertension, obstructive sleep apnoea, stroke, traumatic brain injury, benzodiazepines, proton pump inhibitors, systemic corticosteroids and polypharmacy.
